# Ultrasonography of the Metacarpal/Tarsal-Phalangeal Joints in Healthy Racehorses: Normal Appearance, Breed-Related and Age-Related Features

**DOI:** 10.3390/ani12192657

**Published:** 2022-10-03

**Authors:** Irene Nocera, Caterina Puccinelli, Micaela Sgorbini, Emma Bagnoli, Simonetta Citi

**Affiliations:** 1 Institute of Life Sciences, Sant’Anna School of Advanced Studies, Via Santa Cecilia 3, 56127 Pisa, Italy; 2 Department of Veterinary Sciences, University of Pisa, Viale delle Piagge 2, 56124 Pisa, Italy

**Keywords:** ultrasound, fetlock, horse, cartilage, subchondral bone

## Abstract

**Simple Summary:**

The ultrasound appearance of cartilage thickness and subchondral bone changes according to the age of the horse and the health status of the joints. Specific ultrasound features have been described in different joint diseases in horses. Knowledge of the normal features is thus essential during clinical investigations. The present research evaluates the ultrasound features of metacarpal/tarsal-phalangeal joints in healthy racehorses, according to the age and breed. Twenty-eight racehorses (standardbreds and thoroughbreds) were enrolled and grouped according to age (<5 and ≥5 years old). Fetlock cartilage appearance, thickness and subchondral bone appearance were ultrasonographically assessed. In younger compared to older standardbreds, the cartilage appeared thinner and with a normal ultrasound appearance, which suggests the use of specific references for the ultrasound features of the fetlock.

**Abstract:**

In adult horses, specific ultrasound (US) features and reference values have been reported for the appearance of the joint cartilage and thickness according to the type of joint, such as femoropatellar and tarsocrural. The US appearance of the fetlock has been described in several diseases. The present research evaluates the US features of the metacarpal/tarsal-phalangeal joints in healthy racehorses according to age and breed, since no information is available in the literature. Seventy-one fetlocks in 28 healthy horses (15/28 thoroughbreds and 13/28 standardbreds) were assessed. The horses were grouped as follows: group A < 5 years old vs. group B ≥ 5. A portable ultrasound machine and a linear transducer (5–7.5 MHz) were used. Dorsal metacarpal/tarsal-phalangeal joints were scanned. The US images were reviewed offline in terms of articular cartilage appearance, thickness, and subchondral bone appearance by an experienced observer. Data were reported as the median, minimum, and maximum for cartilage thickness values, and differences between groups were evaluated. Cartilage thickness values were statistically lower in group A than B in the standardbreds, except for the lateral thickness in longitudinal view. No differences were detected in the thoroughbreds within age groups. All of the young standardbreds showed a normal cartilage and subchondral appearance. No statistical differences were found between breeds. Our results highlight the characteristics of the US appearance of metacarpal/tarsal-phalangeal joints specifically in racehorses, with some variations according to age. Since the cartilage can change according to joint growth, age and training activity, the present findings suggest the use of specific references for US features, which are key to correctly evaluating the health of the fetlock.

## 1. Introduction

In horses, the cartilage thickness and subchondral bone ultrasound (US) appearance change according to the age [1] and the different joints [2]. The cartilage thickness is higher in foals and depends on the growth phase, which leads to different degrees of epiphysis ossification [1]. In addition, the cartilage thickness decreases gradually with age, as has been recognised in rats [3], rabbits [4], dogs [5], and humans [6,7].

Cartilage thickness can change according to the different joints: for example, in the femoropatellar joint, it is thicker than in the tarsocrural and intercarpal joints [2]. This is probably related to different biomechanical forces that strongly influence the ossification rate, where high-pressure areas show thicker cartilage [8,9,10,11].

In horse arthropathies, common US features have been extensively described. Irregularity, increased thickness, and linear lesions of the cartilage surface have been reported, while for subchondral bone irregularities, the flattening of the joint profile and non-hyperechogenic appearance have been shown as abnormal [1].

Since the US appearance of the cartilage surface thickness and subchondral bone change markedly in different conditions in the horse, knowledge of the normal features is essential during clinical investigations to recognise potential clinically relevant alterations as early as possible [12].

The current literature in horses underlines how ultrasonography represents a sensitive and valid tool compared to radiography in the early identification of periarticular remodelling and osteochondral lesions, in particular for femoropatellar [13,14], tarsocrural [15,16,17] and metacarpal/tarsal-phalangeal (MCP/MCT) joints [12].

More recent studies have found that US is a valuable field screening tool to detect and monitor early subclinical osteochondrosis lesions, and differentiate physiological from pathological articular events, such as in the femoropatellar joint at the femoral trochlea ridges, both in foals and adult horses [13,18], and in the tarsocrural joint, especially for lesions located on the medial malleolus and distal intermediate ridge of tibia, in adult horses [16].

The US appearance of the fetlock has been described during pathological states [1]. Ultrasound examination seems to match the histological examination of MCP in a population of thoroughbreds, providing significant details on joint morphological features and their variations [19]. To date, there is no research on the features of US in healthy horses according to age and breed.

The aim of the present study was thus to describe the US appearance of metacarpal/tarsal-phalangeal joints in healthy racehorses, and to evaluate potential differences according to age and breed.

## 2. Materials and Methods

### 2.1. Animals

The study was approved by the Institutional Animal Care and Use Committee of the University of Pisa (Prot. N. 6/21), and the owner’s verbal consent was obtained.

Metacarpal/tarsal-phalangeal joints from adult racehorses (thoroughbreds and standardbreds) were evaluated between January 2021 and April 2022. Both thoroughbreds (THs) and standardbreds (STBs) were divided according to age [2,20] into Group A (between 2 and 4 years old) and Group B (≥5 years old). All the horses enrolled in the present study were skeletally mature horses and in active training; they had sound orthopedic [21] and radiographic examinations.

### 2.2. Ultrasound Tecnique

All of the horses underwent US examination of the fetlock. The horses were manually restrained, and the US was performed in the weight-bearing position. The hair over the dorsal fetlock region was not shaved, and alcohol and US gel were applied to provide appropriate contact. Ultrasonography was performed by one experienced operator, in real-time B-mode with a portable US machine (MyLab30Gold, Esaote, Italy) using a multifrequency linear transducer. The following parameters were set: 7.5 MHz frequency, 82% gain, and 6 cm depth for the linear probe. The following anatomical structures were scanned and evaluated: third metacarpal/tarsal distal sagittal ridge and medial and lateral condyles of dorsal metacarpal/tarsal-phalangeal joints. Each structure was evaluated in both longitudinal and transverse sections. All Digital Imaging and Communications in Medicine (DICOM), images and video files were stored and retrospectively reviewed by one experienced operator, using MyLab Desk (Esaote, Genoa, Italy). 

**Figure 1:**
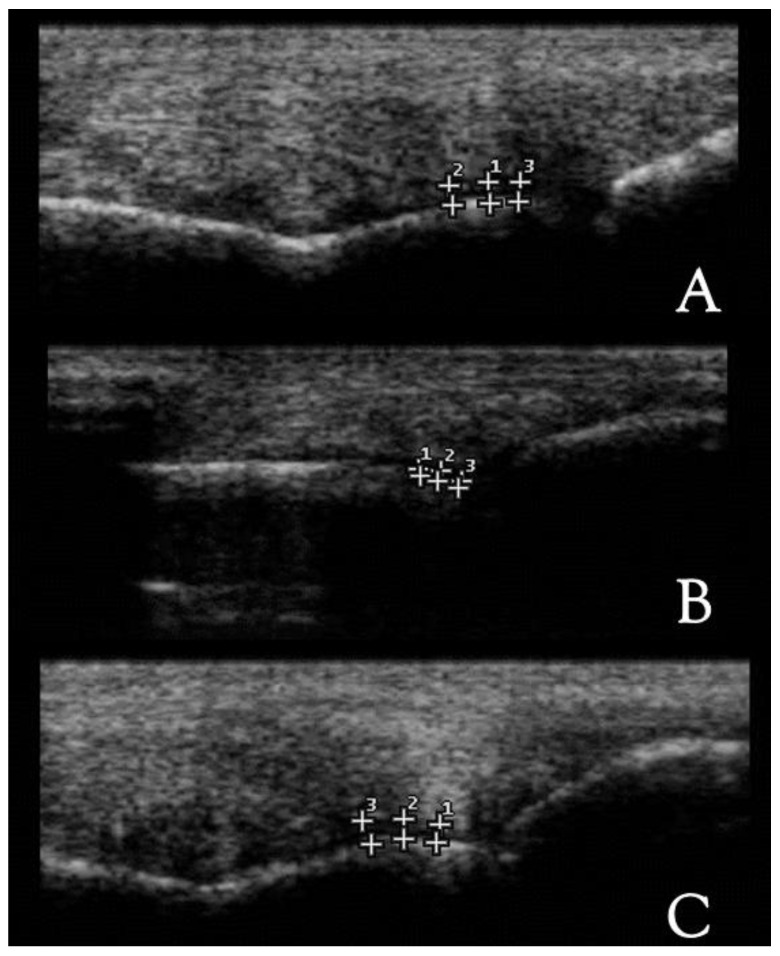
Longitudinal ultrasound views from a thoroughbred MCP joint. (**A**) Dorsal, (**B**) dorso-lateral and (**C**) dorso-medial scans; (1, 2, 3) cartilage thickness consecutive measurements. Proximal is to the left. B-mode, linear probe, 7.5 MHz.

**Figure 2:**
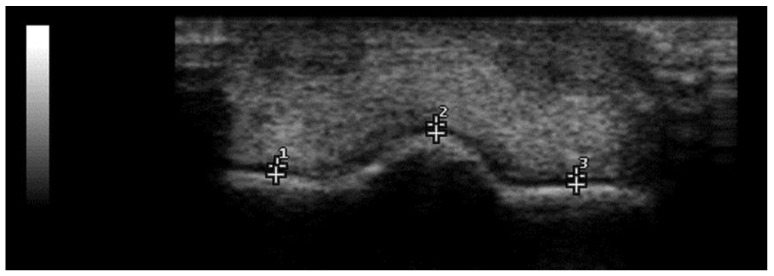
Transversal ultrasound view of thoroughbred MCP joint. (1) Lateral condyle, (2) sagittal ridge and (3) medial condyle. Lateral is to the left. B-mode, linear probe, 7.5 MHz.

From the longitudinal views, three images were obtained from the dorsal, dorso-lateral and dorso-medial perspectives (Figure 1), while one image was obtained from the transversal perspective (Figure 2).

### 2.3. Ultrasound Appearance Evaluation

The following features were evaluated as previously described [1] (Table 1): subchondral bone echogenicity and homogeneity were assessed and judged as normal (e.g., smooth, hyperechogenic interface, producing shadowing and reverberation) or abnormal (e.g., irregular, heterogenic interface); cartilage echogenicity and homogeneity were assessed and judged as normal (i.e., round and smooth in longitudinal section and triangular in cross-section, anechogenic and regular) or abnormal (i.e., irregular and heterogenic), and cartilage thickness was assessed after three consecutive measurements from sagittal ridge, and medial and lateral condyles (interval reference range: 0.7–1 mm [1]. 

### 2.4. Statistical Analysis

The Kolmogorov–Smirnov test was used to evaluate the numerical data distribution. Cartilage thickness showed a non-Gaussian distribution, and the results were reported as median, minimum, and maximum values. The Mann–Whitney test was used to assess differences between Groups A and B, both for STBs and THs, and between breeds.

Regarding cartilage and sub-chondral bone appearance, the Fisher exact test was applied to assess differences (i.e., normal/abnormal) between Groups A and B, both for STBs and THs, and between breeds.

Statistical significance was set at *p* < 0.05, and analyses were performed using a commercial software (GraphPad Prism 9, San Diego, CA, USA).

## 3. Results

### Population Details

A total of 71 fetlock joints from 28 adult racehorses were evaluated. Fifteen out of 28 horses were THs, while 13/28 were standardbreds (STBs). 

Of the STBs, 11/13 were mares and 2/13 were stallions, and the mean body weight was 510 kg (ranging between 460–580 kg); of the THs, 4/15 were mares and 11/15 were stallions, and the mean body weight was 501 kg (ranging between 480–530 kg).

**Figure 3:**
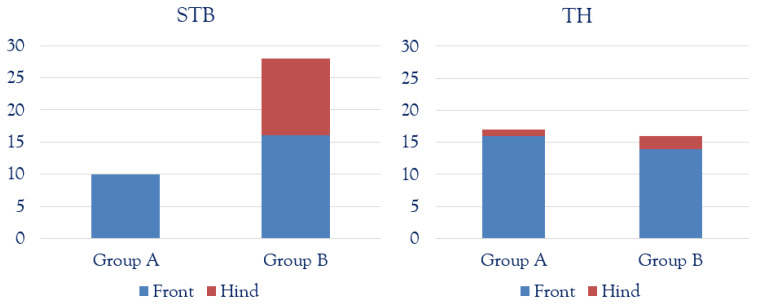
Study population for standardbreds (STBs) and thoroughbreds (THs), in both Group A (2–4 years old) and Group B (>5 years old). Front fetlocks are represented in the blue columns and hind fetlocks in the red ones.

Thirty-eight out of 71 fetlocks from STBs were evaluated (10/38 from Group A and 28/38 from Group B. Thirty-three out of 71 fetlocks from THs were assessed (17/33 from Group A and 16/33 from Group B). For both breeds, front fetlocks were overrepresented compared to the hind fetlock, as shown in Figure 3.

Table 2 and Table 3 report cartilage thickness results for STBs and THs, respectively.

For STBs, statistical differences were detected between Groups A and B, for all the measurements evaluated (longitudinal medial view (*p* = 0.0011), longitudinal dorsal view (*p* = 0.0008), transversal views (*p* < 0.0001)), except for the longitudinal lateral view (*p* = 0.823). Since for all Group A horses, the cartilage appearance was normal, compared to Group B (Group A, abnormal = 0/10; Group B, abnormal = 24/28), the Fisher exact test was not applied. 

**Figure 4:**
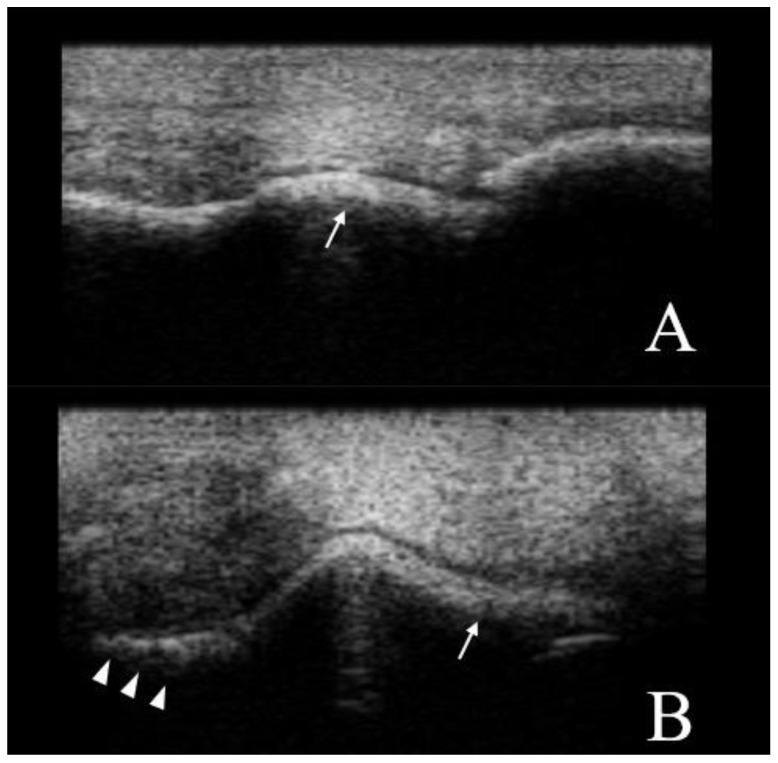
Ultrasound views of a standardbred MCP joint. (**A**) Dorsal longitudinal view, and (**B**) transversal view. The subchondral bone is irregular (arrow heads), showing a sclerotic appearance and shaded subchondral bone-cartilage interface (arrows). Proximal and lateral is to the left. B-mode, linear probe, 7.5 MHz.

A normal US appearance presents a smooth distal metacarpus/tarsus condyle surface, separated by the sagittal ridge which is round in the longitudinal and triangular in the transverse section. The cartilage is anechogenic and homogeneous. The subchondral bone forms a smooth, hyperechogenic interface, and shadowing effect (Figure 1 and Figure 2). In the abnormal fetlocks detected in Group B, the subchondral bone was irregular and showed sclerotic appearance, moreover there was no distinct interface between the subchondral bone and cartilage. The echogenicity of the cartilage was increased, and associated with a heterogeneous appearance (Figure 4). 

For THs, no statistical differences were found between Groups A and B, both in terms of cartilage thickness, and cartilage and subchondral bone appearance (Group A, abnormal = 6/17; Group B, abnormal = 9/16). Abnormal US findings were similar in both groups, which were characterised by focal irregularity of the subchondral bone and discrete heterogeneity of the cartilage interface. Subchondral bone defects or cartilage erosions were not detected. 

No statistical differences were found between breeds, either for cartilage thickness or cartilage and subchondral bone appearance.

## 4. Discussion

Fetlock joint disease is a major cause of lameness and poor performance, with a high economic impact on the racehorse industry. Even young racehorses show MCP cartilage lesions and OA (osteoarthritis) early on [22]. Early diagnosis is thus essential to promptly recognise a potential major issue.

The present study highlighted the characteristics of the US appearance of metacarpal/tarsal-phalangeal joints specifically in standardbred and thoroughbred racehorses, with potential variations according to the different ages.

In younger STB horses, the cartilage appeared thinner (0.5–0.6 mm) compared to older ones (0.8–0.9 mm) and compared to the values reported in the literature for mature horses (0.7–1 mm) [1], except for the longitudinal lateral result (0.5–0.8 mm), which is in line with previously reported values. Our findings are in contrast with those reported for other species, in which cartilage thickness decreased according to age [3,4,5,6,7]. Brunnberg et al. [5] reported a higher cartilage thickness in the talus in young dogs (96 days of age) in which the mean value reported was 0.63 mm, compared to adult dogs (10 years old), with a mean value of 0.33 mm. In younger animals, greater epiphyseal cartilage is normally present, which is progressively replaced by bone tissue through endochondral ossification [11]. However, the epiphyseal ossification rate can be influenced by biomechanical forces and training activities, as reported in horses and other species [1,5,11,12]. This is especially evident in the fetlock joint because of the biomechanical nature of its high-motion condylar joint, which thus receives a high load at high speeds during racing [23].

In our study, younger STBs did not show signs of joint abnormality, such as hyperechoic lines, or anechoic gaps in the subchondral bone [24], reinforcing the hypothesis that a normal thickness variation was detected in the present population. The abnormal US appearance found in all older compared to younger STBs could be related to potential subclinical lesions of the cartilage which may have happened in their previous race training and career, from which they had completely recovered [14]. Moreover, the subchondral trabecular bone adapts to the exercise intensity and type of training, increasing the thickness and mineral density [25]. US alterations detected in our population may thus suggest morphological variations in response to high-speed exercise, as previously reported [19].

Conversely, the TH cartilage thickness in Groups A and B did not differ, and the values were comparable with those reported in the literature for mature horses [1]. Interestingly, 35% of young THs and 56% of older ones showed abnormalities on US joint evaluation. Previous studies reported that 2- and 3-year-old THs might have well-established joint disease and that severe lesions usually occur in horses older than 5 years [26,27]. As shown in humans, clinical signs are highly related to the area extension of the lesions [28], and sometimes no symptoms were detected on clinical and radiographic examination [29,30]. However, our findings support US as being effective in detecting potential joint abnormalities [14]. This is in line with what has been evaluated by previous studies which underline the use of US as a sensitive diagnostic tool to detect early bone irregularities [12]. A recent study compared results from imaging examination of ex vivo model of MCP cartilage and subchondral bone, and it revealed that US images were strictly related to the histological examination, and no statistically significant differences were found between US and radiography [19]. In our study, the sensitivity and the accuracy of US compared to radiography, to detect MCP joint abnormalities, were not evaluated; however, this might represent a stimulus for future studies on this subject.

The abnormal US features recorded in our population are in line with those recently reported by Marsiglia et al. [19]. In their study, irregularities and modifications in the MCP joint cartilage surface and subchondral bone were observed in a population of young THs (median age 3.5 years old). Marsiglia’s [19] findings suggest an anatomical response to normal adaptations during training activities, supporting the theory that the majority of racehorses have some degree of alteration on the articular surface.

Our study showed no differences between STBs and THs for all of the parameters evaluated. However, the low number of horses enrolled might have influenced the statistical analyses, which is one limitation of this study.

Moreover, in our study, the front fetlocks compared to the hind could be regarded as an overrepresentation. Compared to the hind limbs, the forelimbs, and as a consequence fetlock joints, carry more weight, and bear higher impact forces, which results in different load and pressure distribution and in a different cartilage thickness [31]. However, Lee et al. [11] found no significant differences in lateral and medial condyles in the front fetlock (0.87 and 0.81, respectively) compared to the hind ones (0.89 and 0.87, respectively).

## 5. Conclusions

In this study a thinner fetlock cartilage thickness was found in STBs younger than 5 years old; however, no abnormalities in US appearance were detected. These findings support a normal anatomical variability, probably related to different endochondral ossification rates during growth. On the other hand, older STBs showed alterations, which might be related to the morphological adaptations of high biomechanical forces and training.

In THs, US abnormalities were detected both in young and old groups. These results are in line with the previous literature, supporting the evidence that in racehorses, irregularities and modifications might be evident from a young age.

Knowledge of normal US features and variabilities is key to correctly evaluating the health of racehorse fetlocks and promptly recognizing major issues.

## Figures and Tables

**Table 1:** Evaluation criteria applied for the ultrasound appearance assessment.

Anatomical Structure	Evaluation Criteria	
Subchondral bone	Normal	Smooth, hyperechogenic interface, producing shadowing and reverberation
	Abnormal	Irregular, heterogenic interface
Cartilage	Normal	Round and smooth in longitudinal section and triangular in cross-section, anechogenic and regular
	Abnormal	Irregular and heterogenic
Cartilage Thickness	Sagittal ridge	0.7–1 mm
	Medial condyle	
	Lateral condyle	

**Table 2:** Cartilage thickness results reported in millimetres, for standardbreds (STBs), in Group A (A) and Group B (B).

STB		Longitudinal			Transversal		
		Dorso-Lateral Condyle	Sagittal Ridge	Dorso-Medial Condyle	Dorso-Lateral Condyle	Sagittal Ridge	Dorso-Medial Condyle
A	Me (Min–Max)	0.7 (0.5–0.8)	0.6 * (0.5–0.8)	0.6 * (0.5–0.7)	0.5 * (0.4–0.8)	0.5 * (0.4–0.8)	0.5 * (0.4–0.6)
B	Me (Min–Max)	0.8 (0.5–1.7)	0.9 * (0.4–1.7)	0.9 * (0.5–1.8)	0.9 * (0.4–1.3)	0.8 * (0.4–1.1)	0.8 * (0.5–1.9)
*p*-value		0.0856	0.0008	0.0011	<0.0001	<0.0001	<0.0001

* Statistically different (*p* < 0.001).

**Table 3:** Cartilage thickness results reported in millimetres, for thoroughbreds (TH), in Group A (A) and Group B (B).

TH		Longitudinal			Transversal		
		Dorso-Lateral Condyle	Sagittal Ridge	Dorso-Medial Condyle	Dorso-Lateral Condyle	Sagittal Ridge	Dorso-Medial Condyle
A	Me (Min–Max)	0.8 (0.5–1.8)	0.7 (0.5–1.6)	0.8 (0.5–1.5)	0.6 (0.4–1.4)	0.5 (0.4–0.8)	0.8 (0.4–1.8)
B	Me (Min–Max)	0.8 (0.5–1.3)	0.9 (0.5–1.5)	0.9 (0.5–1.4)	0.8 (0.5–1.4)	0.8 (0.4–1.1)	0.9 (0.4–1.1)
*p*-value		0.8230	0.5020	0.7267	0.3006	0.4648	0.3909

## Data Availability

The data presented in this study are available on request from the corresponding author.
